# Comparative Real-World Effectiveness of Fixed-Dose Triple Therapy Regimens in COPD: A Retrospective Cohort Study

**DOI:** 10.3390/jcm15145650

**Published:** 2026-07-18

**Authors:** Rushi Patel, Jacob Thompson, Namra Patel, Abhi C. Lohana, Fnu Versha, Subhash Chander, Viral Patel, Juan Iribarren

**Affiliations:** 1 Department of Internal Medicine, Baptist Hospitals of Southeast Texas, Beaumont, TX 77701, USAiribarrenjuan@hotmail.com (J.I.); 2 Department of Medicine, Paul L. Foster School of Medicine, Texas Tech University Health Sciences Center El Paso, El Paso, TX 79905, USA; 3 Department of Medicine, GMERS Medical College and Hospital, Valsad 396001, Gujarat, India; 4 Department of Internal Medicine, WVU Camden Clark Medical Center, Parkersburg, WV 26101, USA; 5 Department of Pulmonary and Critical Care Medicine, Mayo Clinic, Rochester, MN 55905, USA

**Keywords:** chronic obstructive pulmonary disease, triple therapy, propensity score matching, cohort study, real-world evidence

## Abstract

**Background/Objectives:** Fixed-dose triple therapy is recommended for patients with chronic obstructive pulmonary disease (COPD) at high risk of exacerbations; however, direct comparative effectiveness data between available triple therapy regimens remain limited. We compared real-world clinical outcomes among adults with COPD receiving fluticasone furoate/vilanterol/umeclidinium (FF/VI/UMEC) or budesonide/glycopyrrolate/formoterol (BUD/GLY/FOR). **Methods:** We conducted a retrospective propensity score-matched cohort study using the TriNetX Research Network. Adults with a spirometrically confirmed diagnosis of COPD who received fluticasone furoate/vilanterol/umeclidinium (FF/VI/UMEC) or budesonide/glycopyrrolate/formoterol (BUD/GLY/FOR) between July 2020 and August 2024 were included in the analysis. A 365-day landmark period was applied to establish maintenance therapy, with outcome follow-up beginning 1 year after treatment initiation. Patients were followed for outcomes through August 2025, the date of database analysis. After 1:1 propensity score matching, treatment groups were compared for COPD exacerbations, acute respiratory failure (ARF), hospitalization, and all-cause mortality using RR and Cox proportional hazards analyses. **Results:** In matched cohorts, FF/VI/UMEC was associated with a significantly higher risk of COPD exacerbations (5.6% vs. 3.9%; RR 1.44; 95% CI 1.30–1.59; *p* < 0.001) and ARF (2.5% vs. 1.9%; RR 1.28; 95% CI 1.11–1.46; *p* < 0.001) compared with BUD/GLY/FOR. Time-to-event analyses demonstrated lower event-free probability for exacerbations (HR 1.35; (95% CI 1.21–1.50) and ARF (HR 1.15; (95% CI 1.00–1.32))). All-cause mortality was numerically higher in the FF/VI/UMEC cohort (5.2% vs. 4.7%; RR 1.09; 95% CI 1.00–1.19; *p* = 0.050); however, time-to-event analysis did not demonstrate a statistically significant difference. Hospitalization rates were similar between groups. **Conclusions:** In this large propensity score-matched real-world cohort study, patients receiving BUD/GLY/FOR experienced lower rates of COPD exacerbations and acute respiratory failure than those receiving FF/VI/UMEC. Because of the retrospective observational nature of the analysis, these findings should be considered hypothesis-generating and require confirmation in prospective comparative effectiveness studies.

## 1. Introduction

Chronic obstructive pulmonary disease (COPD) remains a major global public health challenge, affecting more than 390 million people worldwide and accounting for approximately 3.5 million deaths annually, making it the third leading cause of death globally. Despite advances in pharmacologic therapy, COPD continues to impose substantial morbidity, healthcare utilization, and economic burden through progressive respiratory impairment, recurrent exacerbations, and frequent hospitalizations [1,2].

Patients commonly experience dyspnea, chronic cough, sputum production, and exercise intolerance, with many following a course marked by recurrent exacerbations that accelerate lung-function decline, worsen quality of life, and increase long-term morbidity and mortality [3,4,5,6]. Exacerbations are often triggered by viral or bacterial infections, environmental exposures, or aspiration and reflect a complex interplay of airway inflammation, mucus hypersecretion, bronchoconstriction, and ventilation–perfusion mismatch [7]. In moderate-to-severe disease, these events may progress to acute respiratory failure, frequently requiring hospitalization and contributing substantially to mortality [8].

Contemporary COPD management aims to relieve symptoms and prevent exacerbations through optimization of bronchodilation and suppression of airway inflammation [9]. Long-acting muscarinic antagonists (LAMAs) and long-acting β2-agonists (LABAs) remain the foundation of maintenance therapy, while the addition of inhaled corticosteroids (ICSs) further reduces exacerbation risk in appropriately selected patients with persistent symptoms or recurrent exacerbations [10,11,12,13]. Accordingly, international guidelines recommend escalation to fixed-dose ICS/LABA/LAMA triple therapy for patients at increased exacerbation risk despite dual therapy [14,15].

Triple therapy refers to the combined use of an inhaled corticosteroid, a long-acting β2-agonist, and a long-acting muscarinic antagonist, but the available regimens are not pharmacologically or technologically identical. Although available fixed-dose triple therapies combine the same pharmacologic classes, they differ in their constituent molecules, dosing schedules, and inhaler delivery systems. Historically, these regimens have often been considered therapeutically interchangeable based on a presumed “class effect.” However, increasing evidence suggests that clinically meaningful differences may exist despite shared pharmacologic classes [16,17].

ICS molecules differ in receptor affinity, lipophilicity, airway residence time, and immunomodulatory properties, potentially influencing the balance between suppression of airway inflammation and preservation of host defense against respiratory infections [18,19]. Similarly, LABA and LAMA components differ in receptor kinetics and bronchodilator profiles, potentially affecting symptom control, dynamic hyperinflation, and physiologic reserve during respiratory deterioration [20,21].

Differences in inhaler device technology may further influence real-world effectiveness. Fluticasone furoate/vilanterol/umeclidinium (FF/VI/UMEC) is delivered through the Ellipta^®^ dry powder inhaler, whereas budesonide/glycopyrrolate/formoterol (BUD/GLY/FOR) is administered via the Aerosphere^®^ pressurized metered-dose inhaler using co-suspension delivery technology. These platforms differ in inspiratory flow requirements, aerosol generation, and dependence on correct inhalation technique. Such differences may be particularly relevant among older adults and patients with advanced airflow limitation, potentially influencing medication delivery and treatment effectiveness in routine clinical practice [22].

Despite widespread use of fixed-dose triple therapy, comparative effectiveness between individual triple therapy regimens remains incompletely characterized. Most randomized clinical trials have compared triple therapy with dual therapy rather than directly comparing individual triple therapy products [11,12,16,21]. Furthermore, trial populations differ substantially from real-world patients represented in electronic health record (EHR) databases. Clinical trial participants are typically selected using strict eligibility criteria and receive protocol-driven follow-up, standardized inhaler education, and adherence monitoring. Routine clinical populations often have greater comorbidity burden, variable adherence, heterogeneous disease severity, and more complex healthcare utilization. Consequently, real-world comparative effectiveness studies provide complementary evidence by evaluating treatment performance under conditions more representative of everyday clinical practice [20,21].

We hypothesized that differences in pharmacologic properties, inhaler device characteristics, and real-world treatment implementation may translate into clinically meaningful differences in effectiveness between BUD/GLY/FOR and FF/VI/UMEC despite both representing fixed-dose ICS/LABA/LAMA triple therapy. To address this evidence gap, we conducted a retrospective propensity score-matched cohort study using the TriNetX Research Network to compare the effectiveness of these regimens in adults aged ≥45 years with objectively confirmed COPD. We evaluated COPD exacerbations, acute respiratory failure, hospitalization, and all-cause mortality. Ultimately, this study aims to determine whether regimen-specific differences influence clinically important respiratory outcomes and to provide real-world evidence that may help inform individualized treatment selection in COPD.

## 2. Materials and Methods

### 2.1. Study Population

We used the TriNetX Research Network, which included 96 healthcare organizations (HCOs). From the available data, we extracted demographics; diagnoses (ICD-10-CM); procedures (ICD-10-PCS and CPT); medications (Veterans Affairs National Formulary); laboratory tests (LOINC); and healthcare utilization.

The TriNetX Research Network is compliant with the Health Insurance Portability and Accountability Act and General Data Protection Regulation. Since this database aggregates counts and statistical summaries of de-identified data, TriNetX reports that use of the network is granted a waiver of informed consent by the Western Institutional Review Board. The TriNetX analysis was executed on 29 August 2025 (18:57 UTC), representing the data snapshot used for all analyses.

### 2.2. Inclusion and Exclusion Criteria

In our primary cohort, we included patients who were ≥45 years old, had COPD, and had a Forced Expiratory Volume in 1 s (FEV1)/Forced Vital Capacity (FVC) ratio ≤ 0.70. Airflow obstruction was identified using the most recent structured LOINC-coded FEV1/FVC value available within TriNetX. The platform does not distinguish pre- from post-bronchodilator spirometry. Patients with a diagnosis of heart failure (ICD-10-CM I50), pneumonia (ICD-10-CM J18), or asthma (ICD-10-CM J45) were excluded to reduce confounding from overlapping respiratory and cardiopulmonary conditions.

After applying the inclusion and exclusion criteria, two groups were created based on the prescribed inhaler regimen. Cohort 1 included patients who were prescribed a combination of fluticasone, vilanterol, and umeclidinium (FF/VI/UMEC), while cohort 2 included those who were prescribed budesonide, glycopyrrolate, and formoterol (BUD/GLY/FOR). To ensure clear treatment attribution and avoid exposure overlap, patients in cohort 1 were excluded if they were concomitantly prescribed BUD/GLY/FOR, and patients in cohort 2 were excluded if they were concurrently prescribed FF/VI/UMEC. Full cohort query criteria and medication terms (TriNetX UMLS and RxNorm identifiers) are provided in Supplementary Materials.

TriNetX identifies medications by generic name only and does not support querying by brand or combination product name; therefore, cohorts were defined using the individual RxNorm ingredient codes corresponding to each fixed-dose triple therapy product. Cohort assignment required simultaneous presence of all three component medication codes within a single medication order at the same clinical encounter, ensuring that inclusion reflected intentional triple therapy prescribing rather than temporally separated prescriptions of individual inhaler components. The cohort-defined index date corresponded to the first qualifying triple-component order meeting predefined criteria. The TriNetX query was executed after FDA approval of both fixed-dose combination products, and no patient in either cohort carried an index date preceding those approvals.

Laboratory variables were identified using structured LOINC-coded laboratory records available within the TriNetX Research Network, a global federated real-world data and analytics platform [23]. Assay manufacturer information was not available in the analytic output.

### 2.3. Outcomes

The outcomes focused on clinically meaningful respiratory and healthcare utilization events. Acute respiratory failure (ARF) was identified using ICD-10-CM codes J96.0, J96.00, J96.02, J96.20, and J96.22, capturing acute respiratory failure phenotypes including unspecified, hypoxic or hypercapnic, and acute-on-chronic respiratory failure. COPD exacerbations were defined by a recorded diagnosis of COPD with acute exacerbation (ICD-10-CM: J44.1). TriNetX identifies diagnoses from structured ICD-10-CM diagnosis records and does not distinguish primary from secondary diagnosis position within the aggregate analytic output. Hospitalization was identified using the standardized TriNetX inpatient hospitalization construct based on inpatient evaluation and management (E/M) Current Procedural Terminology (CPT) codes. This standardized outcome definition was applied uniformly across all participating healthcare organizations. All-cause mortality was determined based on documented deaths during the study period. Cohort criteria and outcome code lists used in the TriNetX query are provided in Supplementary Materials.

### 2.4. Covariates

To account for differences in baseline characteristics between the two cohorts, we included the following covariates: demographics (age, race), comorbidities (clinical features, respiratory, circulatory, endocrine, and musculoskeletal disorders, neoplasms, skin, subcutaneous, and connective tissue diseases), medications (cardiovascular, central nervous system, and respiratory tract medications, nasal, throat, and dermatological agents, hormones, blood products, antimicrobials), and laboratory records (erythrocytes, hemoglobin, hematocrit, platelets, mean platelet volume, albumin, protein, Brain Natriuretic peptide, N-Terminal-prohormone Brain Natriuretic peptide (NT-proBNP), troponin I, body weight, Body Mass Index (BMI)).

### 2.5. Statistical Analysis

Because this was a retrospective observational study using the TriNetX Research Network, no a priori sample size calculation was performed. All eligible patients meeting the predefined cohort criteria were included. To reduce confounding, we performed propensity score matching using TriNetX’s built-in analysis software (accessed on 29 August 2025). Cohorts were matched 1:1 using greedy nearest-neighbor matching on the propensity score, which incorporated demographics, comorbidities, medications, and baseline laboratory values. Standardized mean differences (SMDs) were used to evaluate the balance of baseline characteristics in matched cohorts, with SMD < 0.1 indicating adequate balance. Although 23,022 patients met eligibility criteria in the smaller treatment cohort before matching, not all patients were retained because propensity score matching only includes patients with an appropriate matched counterpart within the available propensity score distribution. Consequently, the final matched population consisted of 21,626 patients in each cohort. Outcomes were assessed using a prespecified (a priori) 1-year landmark window beginning 365 days after the cohort-defined index event. This landmark period was selected a priori to evaluate long-term comparative effectiveness among patients with established maintenance therapy by allowing sufficient time for sustained treatment exposure while minimizing the influence of early treatment discontinuation, switching, or non-adherence. Patients experiencing the outcome before the landmark were excluded from the corresponding analysis because they were not eligible to enter the landmark risk set at the start of follow-up (details in Supplementary Materials). Risk ratios (RRs) were calculated by comparing event risks between cohort 1 and cohort 2. Time-to-event analyses were performed using Cox proportional hazards models and are reported as hazard ratios (HRs) with 95% confidence intervals (CIs). A two-sided *p*-value < 0.05 was considered statistically significant. Cox proportional hazards regression models were additionally used for comparative analysis because they account for variable patient-level follow-up through right-censoring and estimation of person-time at risk. HRs were considered the primary measure of comparative treatment effect because they appropriately account for differential follow-up through right-censoring and patient-specific time at risk.

Covariate balance after propensity score matching was assessed using SMDs, with an absolute SMD < 0.10 considered indicative of adequate balance. Missing data were not imputed within the TriNetX platform. Cox proportional hazards models were performed using the platform’s built-in survival analysis module, which evaluates time-to-event outcomes within the parameters of the federated TriNetX analytical environment. Detailed cohort definitions, code lists, and TriNetX query parameters are provided in the Supplementary Materials to facilitate reproducibility.

## 3. Results

### 3.1. Baseline Characteristics

In the matched cohorts, the mean age of patients in both cohorts was 67.4 ± 8.9 years. Most baseline characteristics were balanced between cohorts (SMD < 0.1) after matching, as shown in Table 1. Before matching, median follow-up was 475 days in the FF/VI/UMEC cohort and 412 days in the BUD/GLY/FOR cohort. After matching, median follow-up was 550 days for the FF/VI/UMEC cohort and 391.5 days for the BUD/GLY/FOR cohort. Because follow-up duration differed between cohorts, time-to-event analyses using Cox proportional hazards regression were considered the primary comparative estimates, as these appropriately account for variable observation time through right-censoring.

### 3.2. COPD Exacerbations

**Figure 1:**
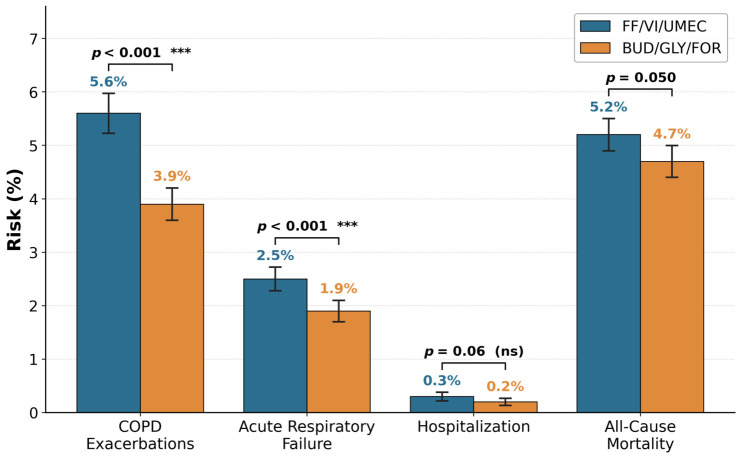
Risk of COPD exacerbation, acute respiratory failure, hospitalization, and all-cause mortality in propensity score-matched cohorts receiving FF/VI/UMEC or BUD/GLY/FOR. Error bars represent 95% confidence intervals calculated using endpoint-specific analytic denominators. *p*-values represent between-group risk-difference tests. Statistical significance for all-cause mortality was not consistently supported across the risk and time-to-event analyses. *** *p* < 0.001; ns, not significant.

Across both treatment groups, 1403 patients experienced a COPD exacerbation, including 805 patients in the FF/VI/UMEC cohort and 598 patients in the BUD/GLY/FOR cohort. Patients treated with FF/VI/UMEC had a 44% higher risk of COPD exacerbation compared with those receiving BUD/GLY/FOR (5.6% vs. 3.9%; RR 1.44 (95% CI 1.30–1.59; *p* < 0.001)) (Figure 1). Time-to-event analysis showed a lower event-free probability in the FF/VI/UMEC cohort (HR 1.35; 95% CI 1.21–1.50).

### 3.3. Acute Respiratory Failure (ARF)

Across both treatment groups, 822 patients experienced acute respiratory failure, including 467 patients in the FF/VI/UMEC cohort and 355 patients in the BUD/GLY/FOR cohort. In the primary analysis, the FF/VI/UMEC cohort had a 28% higher risk of acute respiratory failure compared with the BUD/GLY/FOR cohort (2.5% vs. 1.9%; RR 1.28; 95% CI 1.11–1.46; *p* < 0.001), indicating a modest but statistically significant difference (Figure 1). The time-to-event analysis showed lower event-free probability in the FF/VI/UMEC cohort compared to the BUD/GLY/FOR cohort (HR 1.15; 95% CI 1.00–1.32).

### 3.4. Hospitalizations

The risk of hospitalization did not differ significantly between groups (0.3% vs. 0.2%; RR 1.41; 95% CI 0.98–2.02; *p* = 0.06), with CI crossing unity (Figure 1). Time-to-event analysis likewise demonstrated no significant difference (HR 1.20; 95% CI 0.83–1.72).

### 3.5. Mortality

**Figure 2:**
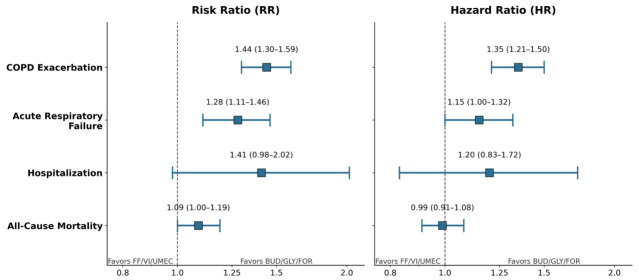
Forest plots showing risk ratios and hazard ratios with 95% confidence intervals for clinical outcomes associated with FF/VI/UMEC compared with BUD/GLY/FOR in the propensity score-matched cohorts. Values greater than 1 indicate a higher occurrence of the adverse outcome in the FF/VI/UMEC cohort.

Compared with the BUD/GLY/FOR cohort, the FF/VI/UMEC cohort demonstrated a numerically higher risk of all-cause mortality (5.2% vs. 4.7%; RR 1.09, 95% CI 1.00–1.19; *p* = 0.050), reaching borderline statistical significance. However, time-to-event analysis did not demonstrate a significant difference (HR 0.99, 95% CI 0.908–1.083) (Figure 2).

## 4. Discussion

In this large propensity score-matched real-world cohort, FF/VI/UMEC was associated with higher risks of COPD exacerbation and acute respiratory failure than BUD/GLY/FOR, whereas hospitalization and time-to-event mortality did not differ significantly between treatment groups. Although cumulative mortality was numerically higher in the FF/VI/UMEC cohort, this finding was not supported by time-to-event analysis. Collectively, these findings suggest consistent differences in respiratory outcomes between the two maintenance regimens while emphasizing the need for prospective comparative effectiveness studies to determine whether these observed associations reflect true treatment-related differences.

Importantly, this study was designed to evaluate comparative effectiveness after maintenance therapy had been established using a prespecified 365-day landmark period. Consequently, the findings should be interpreted as reflecting long-term outcomes among patients receiving sustained maintenance treatment rather than comparative effects immediately following therapy initiation. The concordant associations with higher risks of COPD exacerbations and acute respiratory failure suggest a clinically coherent pattern rather than isolated outcome differences. Frequent exacerbations are recognized markers of disease instability and may predispose patients to subsequent respiratory decompensation through worsening airflow limitation, mucus retention, impaired gas exchange, and respiratory muscle fatigue [5,6,7,8].

The higher risk of acute respiratory failure observed in the FF/VI/UMEC cohort should not be interpreted as evidence of greater progression to critical illness. The present study did not evaluate intensive care unit admission, ventilatory support, or other markers of critical illness. Consequently, progression to advanced respiratory failure requiring noninvasive or invasive mechanical ventilation cannot be inferred from these data. Likewise, although recurrent COPD exacerbations have consistently been associated with accelerated lung function decline and disease progression in previous studies, longitudinal pulmonary function was not assessed in the present analysis. Therefore, our findings should not be interpreted as demonstrating differential rates of physiologic disease progression between treatment groups.

In contrast, hospitalization and mortality did not differ significantly between treatment groups. This apparent discordance likely reflects the multifactorial nature of these outcomes. Hospital admission is influenced not only by disease severity but also by institutional practice patterns, healthcare access, physician judgment, resource availability, and patient-specific social factors. Similarly, mortality reflects numerous competing respiratory and non-respiratory pathways that may dilute differences attributable to maintenance inhaler therapy alone. Future studies incorporating recurrent-event analyses, intensive care utilization, ventilatory support, and cause-specific mortality will help clarify the broader clinical implications of these findings.

### 4.1. Potential Mechanisms Underlying Findings

Several biologically plausible and implementation-related factors could contribute to the observed differences between treatment groups, although none were directly evaluated in the present study. Because this was an observational comparative effectiveness analysis, these considerations should be viewed as hypothesis-generating rather than mechanistic conclusions.

One potential explanation relates to differences between the inhaled corticosteroid (ICS) components. Budesonide and fluticasone furoate differ in lipophilicity, tissue retention, receptor affinity, and local immunomodulatory effects, characteristics that may influence the balance between suppression of airway inflammation and preservation of host defense [22,24]. COPD exacerbations arise through complex interactions among viral and bacterial infections, environmental exposures, mucus hypersecretion, and epithelial injury. Consequently, variation in corticosteroid effects could plausibly influence susceptibility to respiratory events [25,26,27]. Although pneumonia was not evaluated in the present study, previous comparative analyses have reported higher pneumonia risk with fluticasone-containing regimens than with budesonide-containing therapies in COPD populations [28]. Whether differences in infection susceptibility contributed to the present findings cannot be determined and requires prospective investigation. Importantly, these observations should not be interpreted as evidence that one ICS is universally superior, as treatment response remains strongly influenced by individual patient characteristics, particularly inflammatory endotype and blood eosinophil profile [18].

Differences in bronchodilator pharmacology and inhaler delivery may also contribute to comparative effectiveness. Variability in receptor kinetics, duration of bronchodilation, and maintenance of airway caliber throughout the dosing interval could theoretically influence dynamic hyperinflation, respiratory reserve, and susceptibility to acute decompensation [29,30]. In addition, FF/VI/UMEC and BUD/GLY/FOR utilize different inhaler platforms with distinct aerosol characteristics, inspiratory flow requirements, and device-handling techniques. These differences may influence medication deposition and real-world treatment effectiveness, particularly among older adults or patients with advanced airflow limitation who may have difficulty generating adequate inspiratory flow or using inhalers correctly [31,32,33].

Residual confounding remains an important alternative explanation despite extensive propensity score matching. Clinicians may preferentially prescribe one regimen over another based on clinical factors that are incompletely captured within EHR data, including symptom burden, prior exacerbation frequency, radiographic phenotype, inhaler technique, pulmonary rehabilitation participation, or anticipated adherence [34]. Likewise, inflammatory phenotype may influence both treatment selection and treatment response. Patients with eosinophilic COPD generally derive greater benefit from ICS-containing regimens than eosinophil-low or predominantly neutrophilic phenotypes [35,36]. Because eosinophil measurements were unavailable for many patients, confounding related to inflammatory endotype cannot be excluded and represents an important priority for future phenotype-guided comparative effectiveness studies.

When interpreted alongside existing randomized evidence, the present findings should be viewed as complementary rather than contradictory. Randomized trials have consistently demonstrated that both FF/VI/UMEC and BUD/GLY/FOR improve clinical outcomes compared with dual therapy in appropriately selected patients [37,38,39]. In contrast, the present study directly compared these regimens under routine clinical conditions, where differences in adherence, inhaler technique, healthcare utilization, comorbidity burden, and patient heterogeneity are substantially greater than in protocol-driven clinical trials [40]. Accordingly, the observed differences likely reflect the combined influence of pharmacologic, device-related, and real-world implementation factors rather than the isolated effect of any single component. Together, these findings reinforce the complementary roles of randomized efficacy trials and carefully designed real-world comparative effectiveness studies in informing individualized COPD treatment selection.

### 4.2. Clinical Implications and Public Health Relevance

Large randomized trials have firmly established triple therapy as an effective strategy for reducing COPD exacerbations compared with dual therapy; however, direct head-to-head comparisons between available fixed-dose triple therapy regimens remain limited [41]. Recent observational comparisons have not produced uniform results, with some analyses favoring FF/VI/UMEC and others reporting broadly comparable outcomes between regimens [42,43,44]. Differences in study design, treatment-selection criteria, beginning follow-up at treatment initiation, follow-up duration, outcome definitions, and adjustment for confounding may account for the divergent estimates. The present analysis identified higher risks of COPD exacerbation and ARF among patients receiving FF/VI/UMEC, suggesting that clinically relevant regimen-level differences may emerge under real-world conditions.

If confirmed in prospective studies, these findings may inform individualized treatment selection. Preventing COPD exacerbations remains a central therapeutic objective because recurrent events are associated with disease progression, impaired quality of life, and increased healthcare utilization. Acute respiratory failure represents a major form of clinical deterioration that may require escalation of care, including ventilatory support. Reducing these outcomes could decrease emergency department visits, hospitalizations, and downstream healthcare costs, particularly among older adults with multiple comorbidities [45,46].

Importantly, these findings should not be interpreted as evidence that FF/VI/UMEC lacks clinical efficacy. Both regimens remain evidence-based treatments supported by randomized clinical trials. Rather, our results suggest that regimen-specific differences may influence respiratory outcomes in selected real-world populations and therefore support individualized treatment selection rather than assumptions of complete therapeutic equivalence.

With respect to mortality, time-to-event analyses did not demonstrate a statistically significant difference between treatment groups. Although cumulative mortality was numerically higher among patients receiving FF/VI/UMEC, the borderline RR (*p* = 0.050) and CI lower bound of 1.00 warrant cautious interpretation. The apparent difference between the cumulative RR and HR reflects the different statistical quantities estimated by these methods. Whereas the RR compares cumulative event proportions over follow-up, the HR incorporates differences in patient-level follow-up time and right-censoring, providing the more appropriate estimate of relative mortality risk over time in this observational cohort. Accordingly, these mortality findings should be interpreted as hypothesis-generating rather than confirmatory.

COPD mortality reflects numerous competing biological and non-respiratory pathways, while all-cause mortality within EHR datasets remains particularly susceptible to residual confounding and variability in outcome ascertainment [47]. Consequently, the present findings provide stronger evidence for differential respiratory outcomes than for differences in mortality.

### 4.3. Strengths and Limitations

This study has several important strengths. It leveraged a large, geographically diverse federated EHR network encompassing multiple healthcare organizations, enhancing the generalizability of the findings to routine clinical practice. Importantly, cohort identification extended beyond diagnostic coding by requiring objective spirometric evidence of airflow obstruction, thereby improving phenotypic specificity compared with many administrative database studies. Propensity score matching achieved excellent balance across a broad range of demographic characteristics, comorbidities, concomitant medications, and available laboratory variables, substantially reducing measured confounding. Finally, the complementary use of cumulative risk estimates and time-to-event analyses provided a more comprehensive assessment of comparative effectiveness than either analytical approach alone.

Nevertheless, several limitations warrant consideration. As with all retrospective observational studies, residual confounding cannot be eliminated despite rigorous propensity score matching. Although balance was achieved for nearly all measured baseline characteristics, modest residual imbalance remained for hemoglobin (SMD 0.206), hematocrit (SMD 0.131), and serum albumin (SMD 0.219). Importantly, these variables were modestly higher in the FF/VI/UMEC cohort and are generally associated with better hematologic status, nutritional reserve, and overall physiologic health. Consequently, any residual bias arising from these imbalances would be expected to attenuate, rather than explain, the observed excess respiratory risk associated with FF/VI/UMEC. NT-proBNP, by comparison, demonstrated adequate post-matching balance (SMD 0.071).

Several clinically relevant variables were unavailable or inconsistently captured within the TriNetX network and therefore could not be incorporated into the matching process. These included smoking status, vaccination history, inhaler technique, pulmonary rehabilitation participation, medication adherence and persistence, duration of prior inhaled therapy, payer status, baseline eosinophil counts, and detailed spirometric measurements beyond the inclusion criterion. Likewise, available laboratory data did not permit comprehensive characterization of inflammatory endotypes or reliable differentiation between infectious and noninfectious exacerbation triggers. Consequently, residual confounding related to disease severity, inflammatory phenotype, and treatment implementation cannot be excluded despite extensive adjustment for measured covariates.

Median follow-up differed between treatment groups because patients were observed within an open-ended longitudinal EHR framework until outcome occurrence, censoring, or the end of available data. This variability reflects differences in healthcare utilization and EHR availability across participating institutions rather than deficiencies in the propensity score matching procedure, which balances baseline characteristics but does not constrain subsequent observation time. Accordingly, Cox proportional hazards models were selected as the principal comparative analysis because they appropriately account for differential patient-level follow-up through right censoring. Although unequal follow-up may influence cumulative incidence estimates, particularly late during Kaplan–Meier follow-up, hazard ratio estimates explicitly account for differences in follow-up duration through censoring and the timing of events. This distinction likely explains the observed divergence between cumulative mortality estimates and time-to-event mortality analyses. Accordingly, differences between cumulative incidence estimates and time-to-event hazard estimates should be interpreted in the context of unequal follow-up duration and right-censoring, which influence these complementary measures differently.

Outcome ascertainment is also subject to the limitations inherent to EHR-based research. Because outcomes were evaluated using a prespecified 365-day landmark, the analysis reflects conditional longer-term outcomes among patients who remained under observation beyond the treatment-establishment period. Continuous treatment throughout this period was not required by the analytic design and could not be verified in the available TriNetX data. Therefore, treatment interruptions or gaps may have occurred. Importantly, the same landmark definition and outcome ascertainment approach were applied uniformly to both treatment groups, supporting internal comparability. Nevertheless, this design may introduce survivor or selection bias and should not be interpreted as estimating treatment effects from therapy initiation. COPD exacerbations were identified using ICD-10-CM diagnosis codes and therefore may not capture events managed empirically with corticosteroids or antibiotics outside participating institutions, potentially underestimating absolute exacerbation frequency. Similarly, acute respiratory failure was identified using standardized diagnostic codes without independent clinical adjudication. Although identical outcome definitions were applied across both cohorts, variation in institutional coding practices may have resulted in nondifferential outcome misclassification, which would be expected to bias results toward the null rather than exaggerate between-group differences. Baseline exacerbation frequency and recurrent-event burden were likewise unavailable, and hospitalization should be interpreted as an administrative healthcare utilization outcome rather than a direct measure of respiratory severity.

Finally, several limitations are inherent to the TriNetX itself. The platform cannot capture healthcare encounters occurring outside participating organizations, nor can the RxNorm-based medication capture reliably distinguish fixed-dose single-inhaler therapy from concurrent prescribing of individual inhaler components. Consequently, incomplete outcome ascertainment and exposure misclassification remain possible. Despite these limitations, the consistency of the observed respiratory findings across complementary analytical approaches supports the robustness of the overall conclusions while emphasizing the need for prospective comparative effectiveness studies to confirm these associations. No observational study can establish causality; accordingly, the present findings should be interpreted as estimates of comparative effectiveness rather than definitive treatment effects.

### 4.4. Directions for Future Research

Future research should focus on strengthening causal inference and identifying which COPD subgroups derive the greatest benefit from specific fixed-dose triple therapies. Pragmatic prospective comparative effectiveness studies would help reduce confounding by indication while capturing patient-centered outcomes, and when head-to-head trials are not feasible, well-designed real-world approaches (e.g., active-comparator new-user cohorts and target trial emulation) can improve interpretability. Studies should also incorporate biomarker- and phenotype-based stratification, especially baseline and longitudinal blood eosinophils and other type 2 inflammation markers, to clarify corticosteroid responsiveness and the benefit–risk balance across endotypes, alongside infection-risk assessment (e.g., pneumonia indicators, antibiotic exposure). Finally, mechanistic and implementation-focused work should evaluate inhaler device factors and real-world use (technique, inspiratory flow capability, adherence/persistence) and link these to physiologic and imaging phenotypes, while moving beyond time-to-first event to quantify recurrent exacerbations, severe events requiring acute care, post-exacerbation transitions, recovery, and key safety outcomes such as pneumonia and other ICS-associated risks.

## 5. Conclusions

In this large, propensity-score-matched real-world cohort of adults with COPD, budesonide/glycopyrrolate/formoterol (BUD/GLY/FOR) was associated with more favorable respiratory outcomes than fluticasone furoate/vilanterol/umeclidinium (FF/VI/UMEC), particularly with respect to COPD exacerbations and acute respiratory failure (ARF). Although causality cannot be established in this retrospective analysis, these findings suggest that all fixed-dose triple therapies may not necessarily demonstrate equivalent real-world effectiveness and underscore the need for prospective, biomarker-guided comparative effectiveness studies to determine which patients derive the greatest benefit from individual triple therapy regimens.

## Figures and Tables

**Table 1:** Baseline characteristics of the patients before and after propensity score matching.

Baseline Characteristics	Before Propensity Score Matching				After Propensity Score Matching			
	FF/VI/UMEC Cohort (*n* = 58,423)	BUD/GLY/FOR Cohort (*n* = 23,022)	*p*-Value	SMD	FF/VI/UMEC Cohort (*n* = 21,626)	BUD/GLY/FOR Cohort (*n* = 21,626)	*p*-Value	SMD
**Demographics**								
Age at Index (years), mean ± SD	68.3 ± 8.9	67.1 ± 9.1	<0.001 ***	0.135	67.4 ± 8.9	67.4 ± 9.0	0.884	0.001
**Race/ethnicity, *n (%)***								
White	43,175 (73.9)	18,108 (78.7)	<0.001 ***	0.112	17,022 (78.7)	16,857 (77.9)	0.054	0.019
Black or African American	4543 (7.8)	1738 (7.5)	0.275	0.009	1536 (7.1)	1665 (7.7)	0.018 *	0.023
Hispanic or Latino	1380 (2.4)	606 (2.6)	0.024 *	0.017	562 (2.6)	560 (2.6)	0.952	0.001
Asian	1231 (2.1)	291 (1.3)	<0.001 ***	0.066	318 (1.5)	289 (1.3)	0.236	0.011
Unknown Race	8025 (13.7)	2374 (10.3)	<0.001 ***	0.105	2297 (10.6)	2325 (10.8)	0.663	0.004
**Comorbidities, *n (%)***								
Respiratory diseases	45,561 (78.0)	19,473 (84.6)	<0.001 ***	0.170	18,045 (83.4)	18,166 (84.0)	0.115	0.015
Cardiovascular disease	38,734 (66.3)	17,270 (75.0)	<0.001 ***	0.192	15,893 (73.5)	16,027 (74.1)	0.143	0.014
Endocrine, nutritional and metabolic diseases	36,881 (63.1)	16,318 (70.9)	<0.001 ***	0.165	15,085 (69.8)	15,226 (70.4)	0.139	0.014
Diseases of the musculoskeletal system and connective tissue	33,530 (57.4)	14,936 (64.9)	<0.001 ***	0.154	13,822 (63.9)	13,960 (64.6)	0.166	0.013
Neoplasms	18,561 (31.8)	9336 (40.6)	<0.001 ***	0.184	8511 (39.4)	8484 (39.2)	0.790	0.003
Diseases of the skin and subcutaneous tissue	15,550 (26.6)	7192 (31.2)	<0.001 ***	0.102	6757 (31.2)	6772 (31.3)	0.876	0.001
**Baseline Laboratory Values**								
RBCs (10^6^/uL), mean ± SD	4.5 ± 0.7	4.3 ± 0.8	<0.001 ***	0.210	4.5 ± 0.7	4.3 ± 0.8	<0.001 ***	0.196
Hemoglobin (g/dL), mean ± SD	13.5 ± 2.1	13.0 ± 2.4	<0.001 ***	0.219	13.5 ± 2.1	13.0 ± 2.4	<0.001 ***	0.206
Hematocrit (%), mean ± SD	39.3 ± 10.1	38.5 ± 9.1	<0.001 ***	0.082	39.7 ± 9.2	38.5 ± 9.3	<0.001 ***	0.131
Platelets (10^3^/uL), mean ± SD	253.3 ± 86.2	250.0 ± 94.2	<0.001 ***	0.036	253.0 ± 86.1	250.2 ± 93.7	0.005 **	0.032
Platelet mean volume (fL), mean ± SD	9.9 ± 5.5	9.8 ± 6.1	0.013 *	0.025	9.8 ± 3.9	9.8 ± 6.3	0.383	0.011
Serum albumin (g/dL), mean ± SD	4.0 ± 0.5	3.8 ± 0.6	<0.001 ***	0.256	4.0 ± 0.5	3.8 ± 0.6	<0.001 ***	0.219
Serum protein (g/dL), mean ± SD	7.0 ± 0.8	6.8 ± 1.1	<0.001 ***	0.132	7.0 ± 0.7	6.8 ± 1.1	<0.001 ***	0.130
BNP (pg/mL), mean ± SD	168.3 ± 609.0	148.0 ± 300.3	0.085	0.042	146.6 ± 460.2	145.9 ± 295.2	0.942	0.002
NTpBNP (pg/mL), mean ± SD	806.6 ± 3192.8	1187.1 ± 3949.2	<0.001 ***	0.106	912.5 ± 3790.3	1188.6 ± 3960.6	0.031 *	0.071
Troponin I (ng/mL), mean ± SD	0.9 ± 12.7	0.5 ± 4.0	0.115	0.041	0.7 ± 6.3	0.5 ± 4.2	0.195	0.036
Body weight (kg), mean ± SD	80.1 ± 22.5	80.6 ± 23.0	0.006 **	0.026	80.9 ± 23.0	80.6 ± 23.0	0.236	0.014
BMI (kg/m2), mean ± SD	28.1 ± 7.2	28.1 ± 7.4	0.384	0.008	28.3 ± 7.2	28.1 ± 7.3	0.007 **	0.031
**Medications, *n (%)***								
Respiratory Tract medications	50,767 (86.9)	21,133 (91.8)	<0.001 ***	0.159	19,504 (90.2)	19,771 (91.4)	<0.001 ***	0.043
Nasal and Throat agents	47,585 (81.4)	20,604 (89.5)	<0.001 ***	0.230	18,940 (87.6)	19,232 (88.9)	<0.001 ***	0.042
Antimicrobials	39,719 (68.0)	17,752 (77.1)	<0.001 ***	0.206	16,306 (75.4)	16,580 (76.7)	0.002 **	0.030
Cardiovascular medications	45,352 (77.6)	19,554 (84.9)	<0.001 ***	0.188	17,994 (83.2)	18,269 (84.5)	<0.001 ***	0.035
CNS medications	46,394 (79.4)	19,992 (86.8)	<0.001 ***	0.199	18,383 (85.0)	18,655 (86.3)	<0.001 ***	0.036
Follow up (days), Mean ± SD	675.47 ± 665.77	717.08 ± 839.14	-	-	757.99 ± 718.35	666.65 ± 777.59	-	-
Follow up (days), median (IQR)	475 (874)	412 (930)	-	-	550 (965)	391.5 (866)	-	-

Significance symbols: * *p* < 0.05, ** *p* < 0.01, *** *p* < 0.001. The *p*-value compares the two cohorts. An absolute SMD < 0.10 indicates adequate balance. Most covariates achieved adequate balance after matching. Residual imbalance remained for erythrocyte count, hemoglobin, hematocrit, serum albumin, and serum protein. Statistically significant *p*-values despite small SMDs may reflect the large sample size. Abbreviations: FF, fluticasone; VI, vilanterol; UMEC, umeclidinium; BUD, budesonide; GLY, glycopyrrolate; FOR, formoterol; SD, standard deviation; SMD, standardized mean difference; RBC, red blood cells; BNP, brain natriuretic peptide; NTpBNP, N-terminal-prohormone brain natriuretic peptide; BMI, body mass index; CNS, central nervous system; IQR, interquartile range.

## Data Availability

The data used for this study were obtained from the TriNetX Research Network and are not publicly available for direct distribution. Aggregate outputs, cohort definitions, and outcome specifications can be shared upon reasonable request, subject to TriNetX platform policies and institutional access.

## Supplementary Materials

The following supporting information can be downloaded at: https://www.mdpi.com/article/10.3390/jcm15145650/s1, Supplementary Methods S1: TriNetX Cohort and Outcome Definitions; Table S1: Outcome-Specific Post-Landmark Analytic Cohorts.
