# Supplementary material for: Comparative Real-World Effectiveness of Fixed-Dose Triple Therapy Regimens in COPD: A Retrospective Cohort Study
Source: J Clin Med. 2026 Jul 18;15(14):5650. doi: 10.3390/jcm15145650 (PMC13412507; doi:10.3390/jcm15145650)
Supplement: Supplementary file 1 [file jcm-15-05650-s001.zip › jcm-4405165-supplementary.pdf]

Supplementary Materials

Supplementary Methods S1. TriNetX Cohort and Outcome Definitions

Supplementary Methods S1 provides detailed TriNetX cohort definitions, outcome definitions, and analytic parameters used in the study.

TriNetX data freeze

The TriNetX "Compare Outcomes" analysis was generated on August 29, 2025, and results reflect the database state at that time. Eligible index events occurred during a 50-month inclusion period from July 2020 through August 2024.

Cohort definitions (TriNetX query criteria)

Cohort 1: COPD on FF/VI/UMEC

Inclusion criteria: COPD diagnosis (ICD-10-CM J44); age ≥45 years at index; airflow obstruction defined as the most recent $FEV_1/FVC \leq 0.70$ (LOINC 19926-5); and recorded prescriptions for umeclidinium (RxNorm 1487514), fluticasone (RxNorm 41126), and vilanterol (RxNorm 1424884).

Exclusion criteria: recorded prescription for glycopyrrolate (RxNorm 1546438) plus budesonide (RxNorm 19831) plus formoterol (RxNorm 25255), as a combination; and diagnoses of heart failure (ICD-10-CM I50), pneumonia (ICD-10-CM J18), or asthma (ICD-10-CM J45).

Cohort 2: COPD on BUD/GLY/FOR

Inclusion criteria: COPD diagnosis (ICD-10-CM J44); age ≥45 years at index; airflow obstruction defined as the most recent $FEV_1/FVC \leq 0.70$ (LOINC 19926-5); and recorded

prescriptions for glycopyrrolate (RxNorm 1546438), budesonide (RxNorm 19831), and formoterol (RxNorm 25255).

Exclusion criteria: diagnoses of heart failure (ICD-10-CM I50), pneumonia (ICD-10-CM J18), or asthma (ICD-10-CM J45); and recorded prescription for umeclidinium (RxNorm 1487514) plus fluticasone (RxNorm 41126) plus vilanterol (RxNorm 1424884), as a combination.

Index event definition (TriNetX setup)

The index date for each patient was the date of the first qualifying same-encounter triple-component prescription order meeting predefined cohort criteria. All three component medications were required to appear within a single medication order at the same clinical encounter, reflecting intentional combination inhaler prescribing rather than temporally separated prescriptions of individual agents. The TriNetX query was executed after FDA approval of both fixed-dose combination products, and no patient carried an index date preceding those approvals.

Outcome definitions (TriNetX outcomes)

Acute respiratory failure (ARF): ICD-10-CM J96.0, J96.00, J96.02, J96.20, and J96.22.

COPD exacerbation: ICD-10-CM J44.1.

Hospitalization: UMLS:CPT:1013699 (Inpatient or Observation Consultations).

All-cause mortality: TriNetX demographic status recorded as “Deceased”.

Time window (TriNetX setup)

A prespecified 365-day landmark period was applied to evaluate outcomes during established

maintenance therapy. Outcome ascertainment began 365 days after the index date and continued through the available follow-up period, with no fixed end date specified. Using the TriNetX setting “exclude patients with outcome prior to the time window,” patients with the relevant outcome recorded before the start of the post-landmark period, including before the index date or during the initial 365 days, were excluded from that endpoint-specific analysis. This created endpoint-specific post-landmark analytic cohorts comprising patients without a recorded occurrence of the relevant outcome before the start of follow-up. Because the exclusion criterion was applied separately to each endpoint, the final analytic cohort size varied by outcome.

Outcome-specific post-landmark analytic cohorts

The propensity score-matched population initially included 21,626 patients in each treatment cohort. Because the pre-window exclusion was applied separately for each endpoint, the number of patients contributing to each post-landmark analysis varied by outcome.

Table S1. Outcome-specific post-landmark analytic cohorts.

**Outcome**	**FF/VI/UMEC analytic cohort, n**	**BUD/GLY/FOR analytic cohort, n**	**FF/VI/UMEC excluded before window, n**	**BUD/GLY/FOR excluded before window, n**
COPD exacerbation	14,504	15,491	7,122	6,135
Acute respiratory failure	18,865	18,307	2,761	3,319
Hospitalization	20,694	20,534	932	1,092
All-cause mortality	20,633	19,593	993	2,033

Note: Exclusions were endpoint-specific and represent patients with the relevant outcome recorded before the start of the post-landmark outcome window.
